# Crystal structure of bis­[(5-amino-1*H*-1,2,4-triazol-3-yl-κ*N* ^4^)acetato-κ*O*]di­aqua­nickel(II) dihydrate

**DOI:** 10.1107/S1600536814021436

**Published:** 2014-10-04

**Authors:** Victor M. Chernyshev, Anna V. Chernysheva, Raisa S. Abagyan, Victor B. Rybakov

**Affiliations:** a South–Russia State Technical University Prosveschenya, 132, Novocherkassk, Rostov Region, 346428, Russian Federation; b Department of Chemistry, Moscow State University, 119992 Moscow, Russian Federation

**Keywords:** Crystal structure, triazole, 2-(5-amino-1*H*-1,2,4-triazol-3-yl)acetic acid, chelating ligand, nickel coordination compound, crystal structure

## Abstract

The title compound, [Ni(C_4_H_5_N_4_O_2_)_2_(H_2_O)_2_]·2H_2_O, represents the first transition metal complex of the novel chelating triazole ligand, 2-(5-amino-1*H*-1,2,4-triazol-3-yl)acetic acid (*ATAA*), to be structurally characterized. In the mol­ecule of the title complex, the nickel(II) cation is located on an inversion centre and is coordinated by two water mol­ecules in axial positions and two O and two N atoms from two *trans*-oriented chelating anions of the deprotonated *ATAA* ligand, forming a slightly distorted octa­hedron. The *trans* angles of the octa­hedron are all 180° due to the inversion symmetry of the mol­ecule. The *cis*-angles are in the range 87.25 (8)–92.75 (8)°. The six-membered chelate ring adopts a slightly twisted boat conformation with puckering parameters *Q* = 0.542 (2) Å, Θ = 88.5 (2) and ϕ = 15.4 (3)°. The mol­ecular conformation is stabilized by intra­molecular N—H⋯O hydrogen bonds between the amino group and the chelating carboxyl­ate O atom of two *trans*-oriented ligands. In the crystal, the complex mol­ecules and lattice water mol­ecules are linked into a three-dimensional framework by an extensive network of N—H⋯O, O—H⋯O and O—H⋯N hydrogen bonds.

## Abstract 2

The title compound, bis­[(5-amino-1*H*-1,2,4-triazol-3-yl-κ*N* ^4^)acetato-κ*O*]di­aqua)­nickel(II) dihydrate, is the first transition metal complex of 2-(5-amino-1*H*-1,2,4-triazol-3-yl)acetic acid (*ATAA*).

## Chemical context   

**Figure 1:**
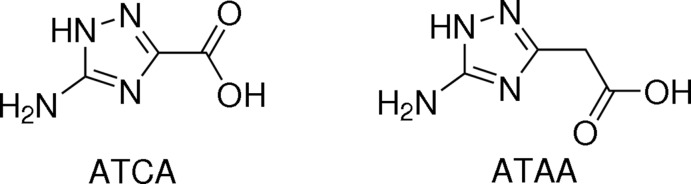
Structural formulas of 5-amino-1*H*-1,2,4-triazole-3-carb­oxy­lic acid (*ATCA*) and 2-(5-amino-1*H*-1,2,4-triazol-3-yl)acetic acid (*ATAA*).

*C*-amino-1,2,4-triazoles are employed as polydentate ligands for the synthesis of coordination compounds with various metals that demonstrate useful spectroscopic, magnetic, biological and catalytic properties (Aromí *et al.*, 2011 ▶; Liu *et al.*, 2011 ▶; Gao *et al.*, 2013 ▶; Hernández-Gil *et al.*, 2014 ▶). Generally, amino­triazoles coordinate metals by either pyridine-type endocyclic nitro­gen atoms or by the amino group (Aromí *et al.*, 2011 ▶; Liu *et al.*, 2011 ▶). Furthermore, amino­triazoles containing substituents with favorably oriented atoms bearing unshared electron pairs (N, S, O *etc*.) can act as chelating polydentate ligands (Biagini-Cingi *et al.*, 1994 ▶; Prins *et al.*, 1996 ▶; Ferrer *et al.*, 2004 ▶, 2012 ▶). 5-Amino-1*H*-1,2,4-tri­azole-3-carb­oxy­lic acid (*ATCA*, Fig. 1 ▶) was found to be a promising chelating ligand for which complexes with various metal cations have been reported recently (Chen *et al.*, 2011 ▶; Sun *et al.*, 2011 ▶; Wang *et al.*, 2011 ▶; Hernández-Gil *et al.*, 2012 ▶; Tseng *et al.*, 2014 ▶). In these complexes, metal cations are chelated by the anions of *ATCA* owing to the formation of coordination bonds with nitro­gen atoms of the triazole ring and the oxygen atom of the deprotonated carb­oxy­lic group.
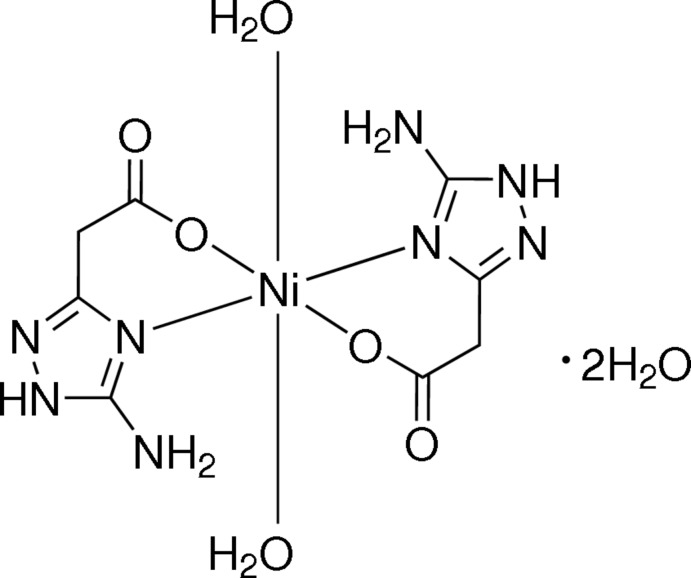


In a continuation of our work on the synthesis and reactivity of amino­triazole carb­oxy­lic acids (Chernyshev *et al.*, 2006 ▶, 2009 ▶, 2010 ▶), we have focused our attention on another chelating ligand, namely 2-(5-amino-1*H*-1,2,4-triazol-3-yl)acetic acid (*ATAA*, Fig. 1 ▶), which can be considered as a homologue of *ATCA*. To the best of our knowledge, *ATAA* or its derivatives have not been studied previously for the synthesis of coordination compounds. Herein, we report the synthesis and crystal structure of an Ni^II^ complex of *ATAA*, the title compound [Ni(C_4_H_5_N_4_O_2_)_2_(H_2_O)_2_]·2H_2_O (1).

## Structural commentary   

**Figure 2:**
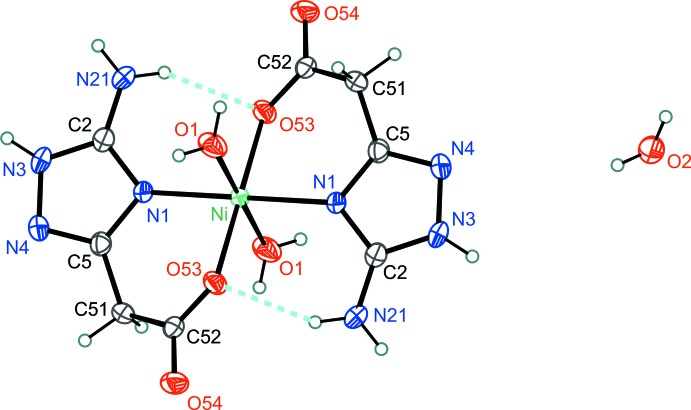
The mol­ecular structure of the title compound, with displacement ellipsoids drawn at the 50% probability level. Intra­molecular N—H⋯O hydrogen bonds are shown as dashed lines. Equivalent atoms are generated by symmetry code −*x*, −*y*, −*z*.

In the mol­ecule of the title complex (1), the Ni^II^ cation is six-coordinated by two bidentate chelating ligands, anions of *ATAA*, and by two water mol­ecules, forming a slightly distorted octa­hedron (Fig. 2 ▶). The *trans*-angles of the octa­hedron are all 180° due to the inversion symmetry of the complete mol­ecule. The *cis*-angles are in the range 87.25 (8)–92.75 (8)°. The third water mol­ecule is not involved in coordination. The anions of *ATAA* coordinate the Ni^II^ cation through the nitro­gen atom N1 of the triazole ring and the oxygen atom O53 of the carboxyl­ate group (Fig. 2 ▶), similarly to the complexes of *ATCA* with various metal cations (Chen *et al.*, 2011 ▶; Sun *et al.*, 2011 ▶; Wang *et al.*, 2011 ▶; Hernández-Gil *et al.*, 2012 ▶). The six-membered chelate ring adopts a slightly twisted boat conformation with puckering parameters of *Q* = 0.542 (2) Å, *Θ* = 88.5 (2), *ϕ* = 15.4 (3)°. The Ni—N1 bond length is 2.051 (2) Å, and the Ni—O1 and Ni—O53 bond lengths are 2.083 (2) and 2.059 (2) Å, respectively, within the normal ranges for other reported Ni^II^ complexes (Lenstra *et al.*, 1989 ▶; Virovets *et al.*, 2000 ▶; Bushuev *et al.*, 2002 ▶; Drozdzewski *et al.*, 2003 ▶; Fan *et al.*, 2010 ▶; Zheng *et al.*, 2011 ▶; Jin *et al.*, 2011 ▶). The amino­triazole fragment N1/C2/N3/N4/C5/N21 is planar (maximum deviation = 0.021 (3) Å for C2), its bond lengths and angles being analogous to complexes of *C*-amino-1,2,4-triazoles with transition metals (Ferrer *et al.*, 2004 ▶; Siddiqui *et al.*, 2011 ▶; Tabatabaee *et al.*, 2011 ▶). The bonds C2—N3 [1.330 (4) Å] and C5—N4 [1.304 (3) Å] are shorter than the bonds C2—N1 [1.342 (3) Å] and C5—N1 [1.365 (3) Å]. The mol­ecular conformation is stabilized by intra­molecular N21—H21*B*⋯O53 hydrogen bonds (Fig. 2 ▶, Table 1 ▶).

## Supra­molecular features   

**Figure 3:**
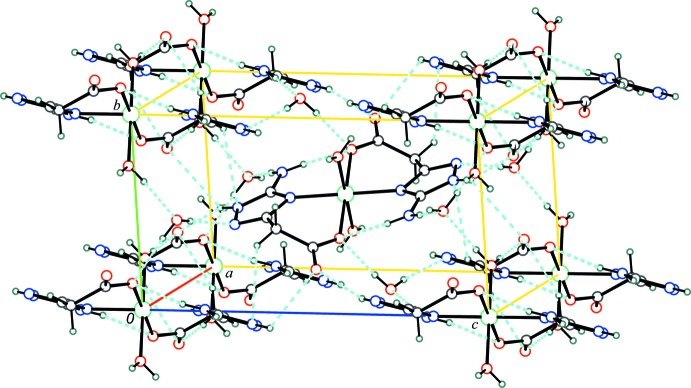
The crystal packing of the title compound viewed along the *a* axis. Hydrogen bonds are shown as dashed lines.

In the crystal, mol­ecules of the complex and lattice water mol­ecules are linked into a three–dimensional framework by extensive N—H⋯O, O—H⋯O and O—H⋯N hydrogen bonds (Table 1 ▶, Fig. 3 ▶).

## Database survey   

More than twenty structures of chelate complexes of 3-substituted 5-amino-1,2,4-triazoles, in which N, O or S atoms of the substituent in the position 3 of the triazole ring play the role of a donor atom, were found in the Cambridge Structural Database (Version 5.35, November 2013 with 2 updates; Thomas *et al.*, 2010 ▶). The database reveals a total of seven structures of coordination compounds of 5-amino-1*H*-1,2,4-triazole-3-carb­oxy­lic acid (*ATCA*) with various metals (Chen *et al.*, 2011 ▶; Sun *et al.*, 2011 ▶; Wang *et al.*, 2011 ▶; Hernández-Gil *et al.*, 2012 ▶; Tseng *et al.*, 2014 ▶; Siddiqui *et al.*, 2011 ▶), six of which are chelate complexes. Coordination compounds of metals with the *ATAA* ligands or its derivatives were not found in the literature.

## Synthesis and crystallization   

**Figure 4:**
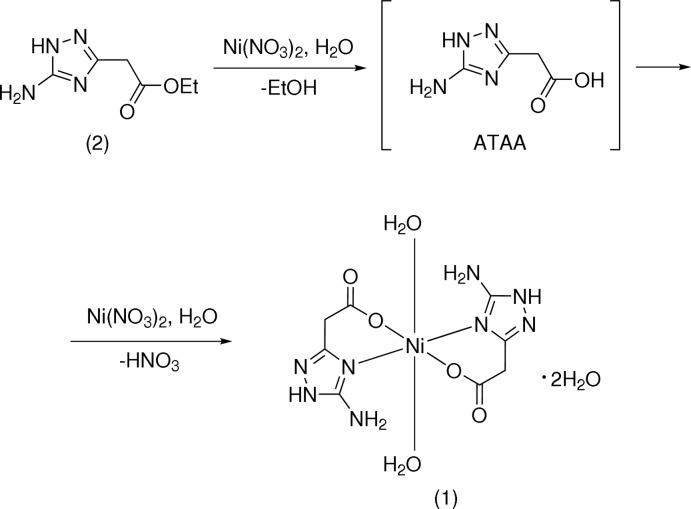
Reaction scheme showing the synthesis of the title compound (1).

All attempts to prepare crystals of complex (1) suitable for X-ray investigation by mixing solutions of *ATAA* or its sodium salt with solutions of Ni^II^ salts were unsuccessful and only microcrystalline precipitates of the sparingly soluble complex were obtained. Crystals of acceptable quality were prepared by slow hydrolysis of ethyl 2-(5-amino-1*H*-1,2,4-triazol-3-yl)acetate (2) in an aqueous solution of nickel nitrate (Fig. 4 ▶). A solution of 0.65 g (3.8 mmol) of compound (2) in water (10 ml) was added to a solution of 0.55 g, (1.9 mmol) of Ni(NO_3_)_2_·6H_2_O in water (5 ml). After standing at room temperature for two weeks, the formed crystals were collected by filtration yielding the target compound (1).

## Refinement   

Crystal data, data collection and structure refinement details are summarized in Table 2 ▶. C-bound H atoms were placed in calculated positions with C—H = 0.97 Å for the CH_2_ group and refined as riding, with *U*_iso_(H) = 1.2*U*_eq_(C). The N,O-bound H atoms that are involved in hydrogen bonds were found from difference Fourier maps. Their distances to the parent atoms were refined to be equal, with a common *U*_iso_(H) value for pairs of related H atoms.

## Supplementary Material

Crystal structure: contains datablock(s) I. DOI: 10.1107/S1600536814021436/wm5066sup1.cif

Structure factors: contains datablock(s) I. DOI: 10.1107/S1600536814021436/wm5066Isup2.hkl

CCDC reference: 1026535

Additional supporting information:  crystallographic information; 3D view; checkCIF report

## Figures and Tables

**Table 1:** Hydrogen-bond geometry (, )

*D*H*A*	*D*H	H*A*	*D* *A*	*D*H*A*
N21H21*A*O2^i^	0.83(2)	2.04(2)	2.876(3)	176(3)
N21H21*B*O53^ii^	0.83(2)	2.19(2)	2.941(3)	151(3)
N3H3O54^iii^	0.83(3)	2.10(3)	2.885(3)	156(3)
O1H1*A*O2^iv^	0.82(2)	1.92(2)	2.739(3)	176(3)
O1H1*B*O54^v^	0.82(2)	1.96(2)	2.780(3)	173(4)
O2H2*A*N4^vi^	0.83(2)	2.09(2)	2.903(3)	164(3)
O2H2*B*O53^vii^	0.83(2)	1.98(2)	2.811(3)	176(3)

Symmetry codes: (i) 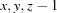; (ii) 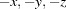; (iii) 
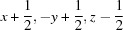
; (iv) 
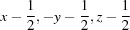
; (v) 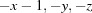; (vi) 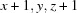; (vii) 
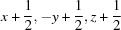
.

**Table 2:** Experimental details

Crystal data	
Chemical formula	
*M*_r_	412.99
Crystal system, space group	Monoclinic, *P*2_1_/*n*
Temperature (K)	295
*a*, *b*, *c* ()	7.6270(17), 7.2603(16), 13.580(3)
()	91.91(2)
*V* (^3^)	751.6(3)
*Z*	2
Radiation type	Ag *K*, = 0.56085
(mm^1^)	0.72
Crystal size (mm)	0.20 0.20 0.20

Data collection	
Diffractometer	EnrafNonius CAD-4
Absorption correction	scan (North *et al.*, 1968 ▶)
*T*_min_, *T*_max_	0.945, 0.958
No. of measured, independent and observed [*I* > 2(*I*)] reflections	1706, 1640, 1215
*R*_int_	0.021
(sin /)_max_ (^1^)	0.638

Refinement	
*R*[*F* ^2^ > 2(*F* ^2^)], *wR*(*F* ^2^), *S*	0.035, 0.077, 1.02
No. of reflections	1640
No. of parameters	140
No. of restraints	3
_max_, _min_ (e ^3^)	0.34, 0.31

Computer programs: *CAD-4 EXPRESS* (EnrafNonius, 1994 ▶), *XCAD4* (Harms Wocadlo, 1995 ▶), *SHELXS97* and *SHELXL2013* (Sheldrick, 2008 ▶), *ORTEP-3 for Windows* and *WinGX* (Farrugia, 2012 ▶).

## supplementary crystallographic information

### Crystal data

**Table:**

	*F*(000) = 428
*M**_r_* = 412.99	*D*_x_ = 1.825 Mg m^−^^3^
Monoclinic, *P*2_1_/*n*	Ag *K*α radiation, λ = 0.56085 Å
Hall symbol: -P 2yn	Cell parameters from 25 reflections
*a* = 7.6270 (17) Å	θ = 10.8–12.9°
*b* = 7.2603 (16) Å	µ = 0.72 mm^−^^1^
*c* = 13.580 (3) Å	*T* = 295 K
β = 91.91 (2)°	Prism, light green
*V* = 751.6 (3) Å^3^	0.20 × 0.20 × 0.20 mm
*Z* = 2	

### Data collection

**Table:**

Enraf–Nonius CAD-4 diffractometer	1215 reflections with *I* > 2σ(*I*)
Radiation source: fine-focus sealed tube	*R*_int_ = 0.021
Graphite monochromator	θ_max_ = 21.0°, θ_min_ = 2.4°
non–profiled ω–scans	*h* = −9→9
Absorption correction: ψ scan (North *et al.*, 1968)	*k* = 0→9
*T*_min_ = 0.945, *T*_max_ = 0.958	*l* = 0→17
1706 measured reflections	1 standard reflections every 60 min
1640 independent reflections	intensity decay: 1%

### Refinement

**Table:**

Refinement on *F*^2^	3 restraints
Least-squares matrix: full	Primary atom site location: structure-invariant direct methods
*R*[*F*^2^ > 2σ(*F*^2^)] = 0.035	Secondary atom site location: difference Fourier map
*wR*(*F*^2^) = 0.077	*w* = 1/[σ^2^(*F*_o_^2^) + (0.0282*P*)^2^ + 0.467*P*] where *P* = (*F*_o_^2^ + 2*F*_c_^2^)/3
*S* = 1.02	(Δ/σ)_max_ < 0.001
1640 reflections	Δρ_max_ = 0.34 e Å^−^^3^
140 parameters	Δρ_min_ = −0.31 e Å^−^^3^

### Special details

**Table:**

Geometry. All s.u.'s (except the s.u. in the dihedral angle between two l.s. planes) are estimated using the full covariance matrix. The cell s.u.'s are taken into account individually in the estimation of s.u.'s in distances, angles and torsion angles; correlations between s.u.'s in cell parameters are only used when they are defined by crystal symmetry. An approximate (isotropic) treatment of cell s.u.'s is used for estimating s.u.'s involving l.s. planes.
Refinement. Refinement of *F*^2^ against ALL reflections. The weighted *R*–factor w*R* and goodness of fit *S* are based on *F*^2^, conventional *R*–factors *R* are based on *F*, with *F* set to zero for negative *F*^2^. The threshold expression of *F*^2^ > 2σ(*F*^2^) is used only for calculating *R*–factors(gt) *etc*. and is not relevant to the choice of reflections for refinement. *R*–factors based on *F*^2^ are statistically about twice as large as those based on *F*, and *R*–factors based on ALL data will be even larger.

### Fractional atomic coordinates and isotropic or equivalent isotropic displacement parameters (Å^2^)

**Table:**

	*x*	*y*	*z*	*U*_iso_*/*U*_eq_	
Ni	0.0000	0.0000	0.0000	0.01840 (14)	
N1	−0.0590 (3)	0.0113 (4)	−0.14834 (14)	0.0215 (5)	
C2	0.0459 (3)	0.0087 (5)	−0.22579 (18)	0.0237 (5)	
N21	0.2095 (3)	−0.0555 (4)	−0.22436 (19)	0.0380 (8)	
H21A	0.278 (3)	−0.036 (5)	−0.2696 (17)	0.040 (7)*	
H21B	0.248 (4)	−0.090 (5)	−0.1692 (15)	0.040 (7)*	
N3	−0.0433 (3)	0.0713 (4)	−0.30469 (18)	0.0307 (6)	
H3	−0.010 (4)	0.083 (4)	−0.362 (2)	0.029 (9)*	
N4	−0.2124 (3)	0.1187 (4)	−0.28023 (17)	0.0289 (6)	
C5	−0.2135 (4)	0.0798 (4)	−0.18651 (19)	0.0218 (6)	
C51	−0.3727 (3)	0.1003 (4)	−0.12754 (19)	0.0242 (6)	
H51A	−0.4200	−0.0215	−0.1160	0.029*	
H51B	−0.4597	0.1678	−0.1668	0.029*	
C52	−0.3496 (3)	0.1962 (4)	−0.02933 (19)	0.0206 (6)	
O53	−0.2044 (2)	0.1806 (3)	0.01848 (14)	0.0248 (5)	
O54	−0.4765 (3)	0.2802 (3)	0.00309 (17)	0.0335 (5)	
O1	−0.1634 (3)	−0.2263 (3)	0.01911 (18)	0.0327 (5)	
H1A	−0.129 (4)	−0.313 (3)	0.053 (2)	0.048 (8)*	
H1B	−0.271 (2)	−0.238 (5)	0.017 (3)	0.048 (8)*	
O2	0.4528 (3)	0.0268 (3)	0.62437 (15)	0.0320 (5)	
H2A	0.556 (3)	0.054 (5)	0.641 (2)	0.039 (7)*	
H2B	0.404 (4)	0.110 (4)	0.591 (2)	0.039 (7)*	

### Atomic displacement parameters (Å^2^)

**Table:**

	*U*^11^	*U*^22^	*U*^33^	*U*^12^	*U*^13^	*U*^23^
Ni	0.0162 (2)	0.0239 (3)	0.0150 (2)	0.0020 (2)	−0.00011 (16)	0.0011 (3)
N1	0.0180 (10)	0.0313 (13)	0.0150 (9)	0.0003 (12)	−0.0003 (8)	0.0021 (12)
C2	0.0253 (13)	0.0265 (14)	0.0193 (12)	−0.0005 (14)	0.0020 (10)	0.0020 (15)
N21	0.0282 (14)	0.063 (2)	0.0236 (13)	0.0098 (13)	0.0102 (10)	0.0131 (13)
N3	0.0322 (14)	0.0449 (16)	0.0152 (12)	0.0033 (12)	0.0051 (10)	0.0053 (11)
N4	0.0275 (13)	0.0400 (16)	0.0190 (12)	0.0058 (12)	−0.0017 (9)	0.0048 (11)
C5	0.0225 (13)	0.0246 (14)	0.0181 (13)	−0.0026 (12)	−0.0009 (11)	0.0006 (11)
C51	0.0161 (13)	0.0340 (17)	0.0224 (14)	0.0006 (12)	−0.0015 (11)	0.0017 (13)
C52	0.0202 (13)	0.0221 (14)	0.0196 (13)	0.0002 (11)	0.0032 (10)	0.0040 (11)
O53	0.0208 (10)	0.0322 (11)	0.0210 (10)	0.0049 (9)	−0.0042 (8)	−0.0041 (9)
O54	0.0238 (11)	0.0486 (13)	0.0282 (10)	0.0106 (10)	0.0032 (9)	−0.0058 (12)
O1	0.0202 (10)	0.0327 (13)	0.0448 (14)	−0.0046 (10)	−0.0041 (10)	0.0108 (11)
O2	0.0309 (11)	0.0371 (14)	0.0280 (11)	−0.0022 (11)	0.0007 (9)	0.0026 (11)

### Geometric parameters (Å, º)

**Table:**

Ni—N1	2.051 (2)	N3—H3	0.83 (3)
Ni—N1^i^	2.051 (2)	N4—C5	1.304 (3)
Ni—O53	2.0590 (19)	C5—C51	1.484 (4)
Ni—O53^i^	2.0590 (19)	C51—C52	1.509 (4)
Ni—O1	2.083 (2)	C51—H51A	0.9700
Ni—O1^i^	2.084 (2)	C51—H51B	0.9700
N1—C2	1.342 (3)	C52—O54	1.238 (3)
N1—C5	1.365 (3)	C52—O53	1.270 (3)
C2—N3	1.330 (4)	O1—H1A	0.822 (19)
C2—N21	1.332 (4)	O1—H1B	0.822 (19)
N21—H21A	0.834 (19)	O2—H2A	0.83 (2)
N21—H21B	0.834 (19)	O2—H2B	0.83 (2)
N3—N4	1.386 (3)		
			
N1—Ni—N1^i^	180.0	H21A—N21—H21B	120 (3)
N1—Ni—O53	87.25 (8)	C2—N3—N4	110.3 (2)
N1^i^—Ni—O53	92.75 (8)	C2—N3—H3	129 (2)
N1—Ni—O53^i^	92.75 (8)	N4—N3—H3	121 (2)
N1^i^—Ni—O53^i^	87.25 (8)	C5—N4—N3	102.5 (2)
O53—Ni—O53^i^	180.00 (13)	N4—C5—N1	114.6 (2)
N1—Ni—O1	92.36 (9)	N4—C5—C51	122.5 (2)
N1^i^—Ni—O1	87.63 (9)	N1—C5—C51	122.9 (2)
O53—Ni—O1	91.63 (9)	C5—C51—C52	116.7 (2)
O53^i^—Ni—O1	88.37 (9)	C5—C51—H51A	108.1
N1—Ni—O1^i^	87.63 (9)	C52—C51—H51A	108.1
N1^i^—Ni—O1^i^	92.37 (9)	C5—C51—H51B	108.1
O53—Ni—O1^i^	88.37 (9)	C52—C51—H51B	108.1
O53^i^—Ni—O1^i^	91.63 (9)	H51A—C51—H51B	107.3
O1—Ni—O1^i^	180.0	O54—C52—O53	122.8 (3)
C2—N1—C5	103.7 (2)	O54—C52—C51	118.2 (2)
C2—N1—Ni	130.69 (17)	O53—C52—C51	119.0 (2)
C5—N1—Ni	123.09 (17)	C52—O53—Ni	130.20 (18)
N3—C2—N21	125.8 (2)	Ni—O1—H1A	120 (2)
N3—C2—N1	108.9 (2)	Ni—O1—H1B	132 (3)
N21—C2—N1	125.2 (2)	H1A—O1—H1B	104 (3)
C2—N21—H21A	123 (2)	H2A—O2—H2B	112 (3)
C2—N21—H21B	115 (2)		
			
C5—N1—C2—N3	−0.3 (3)	Ni—N1—C5—N4	163.8 (2)
Ni—N1—C2—N3	−162.2 (2)	C2—N1—C5—C51	177.4 (3)
C5—N1—C2—N21	−177.1 (3)	Ni—N1—C5—C51	−18.9 (4)
Ni—N1—C2—N21	21.0 (5)	N4—C5—C51—C52	−133.3 (3)
N21—C2—N3—N4	177.1 (3)	N1—C5—C51—C52	49.6 (4)
N1—C2—N3—N4	0.4 (4)	C5—C51—C52—O54	151.7 (3)
C2—N3—N4—C5	−0.2 (3)	C5—C51—C52—O53	−31.5 (4)
N3—N4—C5—N1	0.0 (3)	O54—C52—O53—Ni	162.5 (2)
N3—N4—C5—C51	−177.2 (3)	C51—C52—O53—Ni	−14.2 (4)
C2—N1—C5—N4	0.2 (4)		

Symmetry code: (i) −*x*, −*y*, −*z*.

### Hydrogen-bond geometry (Å, º)

**Table:**

*D*—H···*A*	*D*—H	H···*A*	*D*···*A*	*D*—H···*A*
N21—H21*A*···O2^ii^	0.83 (2)	2.04 (2)	2.876 (3)	176 (3)
N21—H21*B*···O53^i^	0.83 (2)	2.19 (2)	2.941 (3)	151 (3)
N3—H3···O54^iii^	0.83 (3)	2.10 (3)	2.885 (3)	156 (3)
O1—H1*A*···O2^iv^	0.82 (2)	1.92 (2)	2.739 (3)	176 (3)
O1—H1*B*···O54^v^	0.82 (2)	1.96 (2)	2.780 (3)	173 (4)
O2—H2*A*···N4^vi^	0.83 (2)	2.09 (2)	2.903 (3)	164 (3)
O2—H2*B*···O53^vii^	0.83 (2)	1.98 (2)	2.811 (3)	176 (3)

Symmetry codes: (i) −*x*, −*y*, −*z*; (ii) *x*, *y*, *z*−1; (iii) *x*+1/2, −*y*+1/2, *z*−1/2; (iv) *x*−1/2, −*y*−1/2, *z*−1/2; (v) −*x*−1, −*y*, −*z*; (vi) *x*+1, *y*, *z*+1; (vii) *x*+1/2, −*y*+1/2, *z*+1/2.
